# Case Report: Spectrum of interesting ocular manifestations following COVID-19 vaccination: a case series of real-world presentations

**DOI:** 10.3389/fcimb.2023.1243055

**Published:** 2023-09-15

**Authors:** Iqbal Tajunisah, Shao Sze Tan, Irina Effendi-Tenang, Amir Samsudin, Kiet-Phang Ling, Wee Yong Tan, Sunder Ramasamy, Kenneth Choong Sian Fong

**Affiliations:** ^1^ University of Malaya Eye Research Centre (UMERC), Department of Ophthalmology, Faculty of Medicine, University of Malaya, Kuala Lumpur, Malaysia; ^2^ Department of Ophthalmology, Oasis Eye Specialists, Johor Bahru, Malaysia; ^3^ Department of Neurology, Thomson Hospital, Petaling Jaya, Malaysia; ^4^ Department of Ophthalmology, Oasis Eye Specialists, Kuala Lumpur, Malaysia

**Keywords:** COVID-19 vaccination, retinal pigment epitheliitis, acute multifocal placoid pigment epitheliopathy, multifocal choroiditis, herpes zoster uveitis

## Abstract

**Purpose:**

We report the ocular findings that patients experienced after receiving the coronavirus disease 2019 (COVID-19) vaccination in three different eye centers in Malaysia.

**Observations:**

A total of four cases were reported. Three patients received the Pfizer-BioNTech vaccine, while the other received the Oxford AstraZeneca type. Ocular symptoms occurred after the first vaccine dose in two patients and after the second vaccine dose in the other two. Three out of four patients required active treatment for their vision complications postvaccination. The first patient had acute-onset retinal pigment epitheliitis within 3 h of vaccination and was treated conservatively. The second patient developed unilateral choroidal neovascularization 3 days after vaccination and required intravitreal antivascular endothelial growth factor injection. The third patient presented with bilateral acute multifocal placoid pigment epitheliopathy a week after vaccination and responded to intravenous methylprednisolone. The fourth patient presented with herpes zoster infection and unilateral anterior nongranulomatous uveitis 2 weeks after vaccination and was treated with oral acyclovir and topical corticosteroids. All patients reported some amount of visual recovery.

**Conclusions and importance:**

Visual symptoms and various ocular adverse events can occur following COVID-19 vaccination, which warrants further investigation and urgent intervention if necessary. We would suggest patients receiving the COVID-19 vaccination be aware of possible ocular complications and report any symptoms, regardless of severity.

## Introduction

COVID-19, or severe acute respiratory syndrome coronavirus 2 (Sars-CoV-2) emerged as a global pandemic in December 2019 (Lu et al., 2020). The COVID-19 Pfizer-BioNTech vaccine was recommended as an emergency measure by the United States Food and Drug Administration (FDA), with the vaccine being administered intramuscularly in two separate doses, at least 21 days apart (FDA, 2021). The Pfizer-BioNTech (BNT162b2) COVID-19 vaccine is a form of mRNA vaccine that encodes the viral spike protein of COVID-19 and is generally well tolerated in phase III clinical trials (Polack et al., 2020). The Oxford AstraZeneca (ChAdOx1 nCoV-19) COVID-19 vaccine is another type of vaccine given, which is an adenoviral vector vaccine. Both vaccines are generally safe, with short-term mild to moderate side effects and low incidence of serious adverse events (Polack et al., 2020; Knoll and Wonodi, 2021).

Among the serious adverse events reported following administration of the vaccines include anaphylaxis (COVID C, Team R, 2021) and, on a rare occasion, Guillan–Barre syndrome causing neurological complications (Waheed et al., 2021). Other adverse effects reported following COVID-19 vaccines include herpes zoster infection (Bostan and Yalici-Armagan, 2021; Furer et al., 2021) and orofacial involvement such as Bell’s palsy and facial swelling (Cirillo, 2021). Vaccine-induced thrombocytopenia and thrombosis were also reported following administration of ChAdOx1 nCoV-19 (AstraZeneca) vaccine (Pavord et al., 2021; Salih et al., 2021; Wang et al., 2021). From an ophthalmology perspective, various ocular complications have been reported following administration of either the COVID-19 Pfizer-BioNTech or Oxford AstraZeneca vaccine. These include reactivation of herpetic keratitis and varicella zoster corneal endotheliitis (Li et al., 2021), episcleritis (Pichi et al., 2021 anterior scleritis (Pichi et al., 2021), acute macular neuroretinopathy (Pichi et al., 2021), paracentral acute middle maculopathy (Pichi et al., 2021), subretinal fluid (Pichi et al., 2021), and Vogt–Koyanagi–Harada syndrome (Koong et al., 2021; Saraceno et al., 2021).

We report a case series of four patients with various ocular manifestations following the administration of the COVID-19 vaccine, which may or may not be vaccine-related. These include acute retinal pigment epitheliitis, choroidal neovascularization, acute multifocal placoid pigment epitheliopathy, and acute anterior uveitis with herpes zoster infection.

## Method

### Case series

#### Case 1

A 36-year-old male doctor with no underlying medical illness or allergies was administered the first dose of the COVID-19 vaccine (Cominarty, BNT162b2, Pfizer-BioNTech) in a tertiary hospital in Malaysia. He presented to the ophthalmology clinic with left eye metamorphopsia 10 days after vaccination. He claimed to have bilateral metamorphopsia with generalized body aches about 3 h postvaccination. His myalgia and backache resolved after 1 day, and his right eye metamorphosia resolved after 3 days. However, the metamorphopsia in his left eye persisted. Prior to this episode, he did not have any ocular symptoms. There was no eye pain, redness, or photophobia in either eye. No viral-like illness or symptoms, such as cough, upper respiratory tract illness, or fever, were reported before or after his vaccination. Systemic examination was unremarkable. There was no history of close contact with established COVID-19-positive individuals.

**Figure 1:**
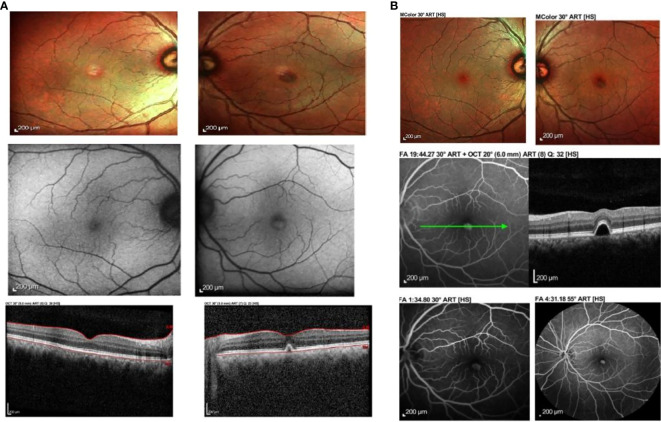
**(A)** Fundus photograph, autofluorescence and ocular coherence tomography findings at 10 days post vaccination. There is left eye yellowish raised elevation and hyperautofluorescence at the fovea with dome-shaped elevation of the RPE at the fovea causing disruption of the ellipsoid zone. No subretinal fluid was noted. **(B)** Fundus photograph, ocular coherence tomography (OCT), fundus fluorescein angiography (FFA) and indocyanine green angiography (ICGA) of the same patient at 15 days post vaccination showed improvement clinically. The yellowish elevated lesion at the fovea had reduced in size, corresponding to the reported improvement in symptoms. The lesion from OCT was still present. There was hyperfluroescence at the macula in venous phase of FFA and hypocyanescence at the macula on ICGA.

His presenting visual acuity was 6/12 OU unaided. The refractive assessment showed bilateral low myope of −0.75D and mild astigmatism of −0.25D in the left eye, with a normal amplitude of accommodation 7.5D OU. The best corrected visual acuity was 6/6 OU. Left-eye metamorphopsia was noted on the Amsler chart, mostly around the central and paracentral regions. There was no central scotoma, macropsia, or micropsia. The anterior segment examination via slit lamp biomicroscopy was unremarkable. There was no evidence to suggest ocular inflammation. Fundus examination of the left eye showed a well-circumscribed, yellowish-raised elevation superior to the fovea. No subretinal fluid, exudates, drusen, or hemorrhages were seen. Examination of the right eye was unremarkable (**Figure 1A**).

Ocular imaging, including spectral-domain OCT, fundus photography, and autofluorescence, was obtained via Heidelberg Spectralis ^®^ HRA+OCT (Heidelberg Engineering, Heidelberg, Germany), which showed a dome-shaped hyper-reflective elevation at the left fovea involving the photoreceptor layer and causing disruption of the ellipsoid zone. Fundus autofluorescence showed hyperautofluorescence at the foveal region that corresponded clinically to the location where the yellowish-white lesion was observed at the macula (**Figure 1A**). These findings were suggestive of acute retinal pigment epithelitis (ARPE).

At 15 days postvaccination, the patient reported some improvement of the metamorphopsia in his left eye. Although it was still present, the symptoms had reduced in intensity, and the visual acuity had improved to 6/6 OU. Fundus examination showed a slight improvement in the size of the lesion at the macula. OCT findings demonstrated a similar lesion at the fovea involving the retinal pigment epithelium. A fundus fluorescein angiography (FFA) and indocyanine green angiography (ICGA) were performed. The former demonstrated hyperfluorescence at the fovea in the venous phase, corresponding to the fundus findings and OCT images. There was no evidence of leakage, pooling, or neovascularization. ICGA showed hypocyanescence at the corresponding area on the fovea, with a hypocyanescent area noted at the inferonasal vessel arcade in the venous phase. No polyps, branching vascular networks, or evidence of choroidal neovascularization were seen (**Figure 1B**).

At 1-month postvaccination, his symptoms improved with minimal residual metamorphopsia on the Amsler grid with a reduction in the area involved. His visual acuity was 6/6 OU. However, the anatomical lesion persisted with residual pigment epithelial defect (PED).

We diagnosed his ocular symptoms as possibly vaccine-related, and our patient subsequently opted not to continue with the second dose of his vaccine.

#### Case 2

**Figure 2:**
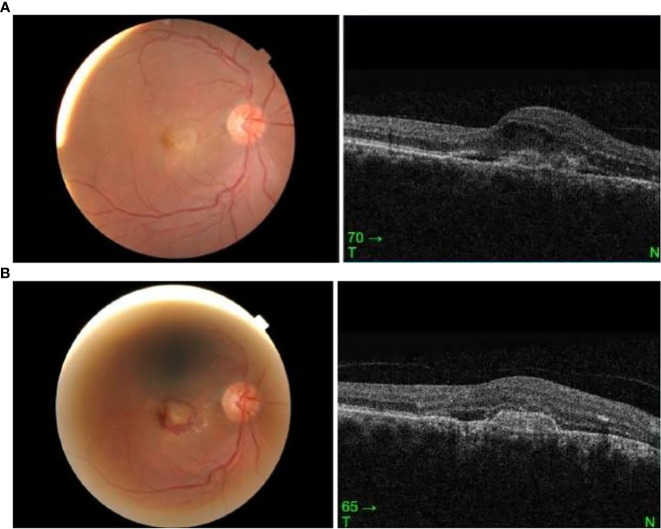
**(A)** Right eye fundus and OCT macula on presentation, 3 days post vaccination. **(B)** Right eye fundus and OCT macula 2 weeks post vaccination.

A 68-year-old Malay man presented with sudden-onset right-eye central blurring of vision 3 days after receiving his second dose of COVID-19 vaccination (Cominarty, BNT162b2, Pfizer-BioNTech). He had no underlying medical illness and no significant past ocular history. He did not have any ocular symptoms after his first dose of the vaccine. His presenting visual acuity was 6/12 OD and 6/6 OS. The anterior segment examination was unremarkable. Fundus examination showed an orange-round lesion at the macula. There was no drusen or evidence of age-related macular degeneration, and no myopic changes. Ocular coherence tomography (Topcon Spectral Domain OCT) showed loss of foveal contour with the presence of intraretinal and subretinal fluid, and a fibrovascular pigment epithelial defect (**Figure 2A**). A diagnosis of presumed choroidal neovascularization was made, based upon clinical and OCT findings. His condition did not improve upon subsequent review 11 days later, and intravitreal ranibizumab at 0.5 mg/0.05 ml was administered in his right eye. Following intravitreal ranibizumab injection 1 week later, his right-eye vision remained the same at 6/12 best-corrected. Examination of the posterior segment showed hemorrhagic changes at the macula. OCT macula revealed resolution of intraretinal fluids; however, the subretinal fluid was similar in size and the fibrovascular PED was more defined (**Figure 2B**).

#### Case 3

**Figure 3:**
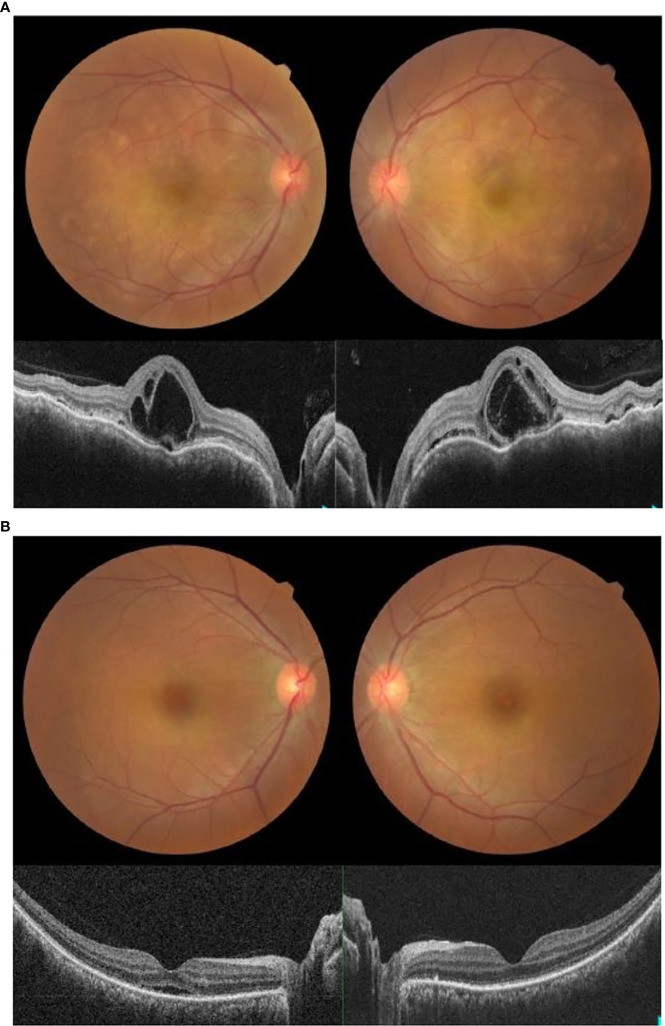
**(A)** Fundus photo and OCT macula showing bilateral placoid lesions in the choroid with macula edema on presentation. **(B)** Fundus photo and OCT macula showing resolution of bilateral placoid lesions and macula edema one week post-treatment with intravenous methylprednisolone.

A 50-year-old Malay woman with no underlying medical illness presented with bilateral blurring of vision 2 weeks after receiving her second dose of COVID-19 vaccination (Cominarty, BNT162b2, Pfizer-BioNTech). Her blurring of vision started 1 week after vaccination and did not improve over time. Her visual acuity was 6/18 OD and 6/12 OS. The anterior segment examination was unremarkable, with no evidence of anterior segment inflammation. Posterior segment examination showed bilateral fibrinous placoid lesions in the choroid with severe inner and outer macula edema (**Figure 3A**). Visual evoked potential and optic nerve conduction studies were normal. The systemic examination was otherwise unremarkable. Blood investigations, including complete blood count and urine microscopy, were not suggestive of infection or other underlying cause. Serology for syphilis, human immunodeficiency virus (HIV), and investigations for tuberculosis were negative. Her COVID-19 PCR test was also negative. She underwent magnetic resonance imaging (MRI) of the brain and orbit which were normal. A diagnosis of acute multifocal placoid pigment epitheliopathy was made based on clinical and investigational findings.

She received intravenous methylprednisolone at 1 mg/kg for 5 days, starting on the day of presentation. Two days later, after intravenous methylprednisolone, her visual acuity improved to 6/9 OU. There was a marked reduction in the placoid lesions and macula edema. Upon subsequent review, her macula edema and placoid lesions resolved 4 days later. A week after being given intravenous methylprednisolone, her symptoms resolved. Her visual acuity was 6/6 OU, with no macula edema or placoid lesions seen both clinically and on ocular imaging (**Figure 3B**).

#### Case 4

A 32-year-old Malay woman who is a housewife with an underlying history of anxiety, panic disorder, and depression received her first dose of Oxford AstraZeneca ChAdOx1 COVID-19 vaccination. Two weeks after her vaccination, she developed multiple skin rashes, vesicles, and crusty lesions over the right side of her face, respecting the midline and involving the tip of the nose. A week later, following the onset of the rash, she developed sudden-onset redness, itchiness, and mild blurring of vision in her right eye. There was also right periorbital swelling, which resolved after 2 days. No photophobia, flashes, floaters, scotoma, or metamorphopsia were noted. She had no underlying medical illnesses other than those mentioned. There was no history to suggest any immunocompromised state. Her visual acuity was 6/12 unaided and 6/6 pinhole OU. An ocular examination showed the presence of a circumciliary injection in her right eye. Corneal sensation was intact and equal bilaterally. There was no evidence of cornea epithelial, stromal, or endothelial involvement. Intraocular pressure was 14 mmHg OU. The anterior chamber showed cell activity at 2+ with no keratic precipitates, hypopyons, or fibrinous reactions. Fundus examination was otherwise unremarkable, with normal optic disc and macula. There was no evidence of posterior segment inflammation such as vitritis, retinitis, choroiditis, or vasculitis. Examination of the left eye was normal. She was treated for an underlying herpes zoster infection with right acute nongranulomatous anterior uveitis. The diagnosis was made clinically. She was treated with oral acyclovir at 800 mg five times a day, together with topical tobramycin at 0.3% and dexamethasone at 0.1% 4-hourly in her right eye. Following treatment, the uveitis and skin lesions improved by two weeks and completely resolved at the 1-month follow-up.

## Discussion

In our case series (**Table 1**), all four patients had ocular symptoms that occurred within hours to weeks after receiving the first dose of COVID-19 vaccination. All were otherwise asymptomatic prior to their vaccination.

**Table 1:** Onset, clinical diagnosis, and treatment of all four patients who developed ocular adverse effects following the first dose of COVID-19 vaccination.

Case	Vaccine	Onset	Diagnosis	Treatment
1	Pfizer-BioNTech Cominarty	3–4 h	Acute retinal pigment epitheliitis	Conservative
2	Pfizer-BioNTech Cominarty	3 days	Choroidal neovascularization (presumed)	Intravitreal ranibizumab 0.5 mg/0.05 ml
3	Pfizer-BioNTech Cominarty	2 weeks	Acute multifocal placoid pigment epitheliopathy	Intravenous methylprednisolone 1 mg/kg × 5 days
4	Oxford AstraZeneca ChAdOx1	2 weeks	Herpes zoster ophthalmicus	Oral acyclovir 800 mg 5 times/day Topical tobramycin at 0.3% and dexamethasone at 0.1% 4-hourly

The diagnosis of ARPE in case 1 was made clinically based on a history of acute-onset central metamorphopsia together with the examination findings over the macula. ARPE is a rare inflammatory condition involving the retinal pigment epithelium (Krill and Deutman, 1972). The exact etiology and pathogenesis of acute retinal pigment epitheliitis remains unknown. It is more common in young adults, self-limiting, and associated with good visual recovery (Krill and Deutman, 1972). Clinical features include acute-onset painless central scotoma due to involvement of the retinal pigment epithelium at the macula (Friedman, 1975). Spectral domain ocular coherence tomography (SD-OCT) in cases of ARPE demonstrates abnormal reflectivity in the RPE inner layer with disruption of the photoreceptor ellipsoid zone and undulation of the retinal pigment epithelium (RPE) (Cho et al., 2011; Kim et al., 2011). These clinical findings correlate with our patient’s clinical presentation. Our patient also reported improvement upon follow-up at 1 month, similar to the good visual recovery following the natural course of the disease, which is self-limiting (Krill and Deutman, 1972).

Case 2 described a choroidal neovascularization (CNV) with macula edema that occurred within 3 days of vaccination. Inflammatory cytokines, especially IL-6 and IL-8, have been found to be significantly associated with the volume of macula edema in choroidal neovascularization (Miao et al., 2012). There is an expression of tissue factor by macrophages in CNV, which is stimulated by monocyte chemotactic protein secreted by RPE cells (Grossniklaus et al., 2002). Both RPE and macrophages also secrete VEGF, which induces choroidal neovascularization (Grossniklaus et al., 2002). Wang et al. reported that the upregulation of VEGF by RPE cells via ROS-dependent activation of β-catenin signaling was induced by TNF-α (Wang et al., 2016). The exact mechanism of CNV formation in our patient remains unclear, but there could be a possible association with the active inflammatory response induced by the vaccine.

As for case 3, a diagnosis of acute multifocal placoid pigment epitheliopathy (AMPPE) was made based on the clinical findings. It is characterized by choriocapillaritis with secondary ischemia of the overlying RPE and outer retina, manifested as placoid lesions. The exact etiology of AMPPE is unknown. It is hypothesized that the primary insult occurs at the level of inner choroid/choriocapillaris with retinal changes occurring secondarily (Burke et al., 2017; Dolz-Marco et al., 2017). These inflammatory lesions can then present as multiple yellow-white placoid subretinal lesions in the posterior pole, as shown in case 3. For AMPPE, the placoid lesions usually resolve in most cases with vision recovery within a few months. However, in view of the centromacular involvement with significantly reduced visual activity, we decided to treat our patient with intravenous corticosteroids for more rapid regression and a possible likely vaccine-induced inflammatory etiology. Following treatment, her visual acuity improved during follow-up.

We also considered other differential diagnoses for case 3, which include multifocal choroiditis and Vogt–Koyanagi–Harada (VKH) syndrome. Multifocal choroiditis has been reported, with the onset of symptoms occurring 1 week following COVID-19 vaccination, with improvement following oral corticosteroids (Goyal et al., 2021). Another possibility is VKH syndrome following COVID-19 vaccination, in which several case reports suggest a causal relationship between the two (Koong et al., 2021; Saraceno et al., 2021). In those patients developing VKH post-COVID-19 vaccination, the condition responded well with oral or intravenous methylprednisolone (Koong et al., 2021; Saraceno et al., 2021). For case 3, VKH remains a possible differential diagnosis. However, clinically, there was no evidence of bilateral granulomatous panuveitis, which is usually seen in an acute episode of VKH, or sunset-glow fundus, Dalen–Fuchs nodules, which suggest chronicity and possible reactivation following vaccination. There was no fundus fluorescein angiogram performed, which may aid in confirming this diagnosis.

It is interesting to note that both cases 1 and 3 received the same type of vaccine. In both patients, the common site of involvement is the retinal pigment epithelium and outer retina spaces. In case 1, the RPE was mainly involved, and the RPE defect persisted weeks postvaccination. However, the onset of symptoms differed between the two; one had visual symptoms within hours after receiving his vaccine, while in case 3, the occurrence was a week later. This difference in the severity of symptoms between the two cases may or may not correspond to the fact that case 1 received only one dose of vaccination as compared to case 3, who had completed her second dose of the vaccination. Case 4 had acute nongranulomatous anterior uveitis following a herpes zoster infection. Herpes zoster infection has been reported following the administration of the COVID-19 vaccine (Bostan and Yalici-Armagan, 2021; Furer et al., 2021). The anterior uveitis was likely secondary to herpes zoster infection instead of the vaccine. There have been reports of anterior uveitis occurring following other forms of live vaccines, such as varicella zoster vaccine (Naseri et al., 2003) and rubella vaccine (Ferrini et al., 2013); however, to date there is no direct correlation with the COVID-19 vaccine.

Our case series has its limitations. All the ocular complications are possibly related to the vaccination, although there is no definite causal relationship. In our case series, all the various presentations were related to different degrees of ocular inflammation. We presume that there could be a possibility of vaccine-mediated immune response leading to some increase in inflammatory mediators, which could have adverse effects on the eye.

## Conclusion

Visual symptoms and various ocular adverse events can occur following COVID-19 vaccination, which warrants further investigation and urgent intervention if necessary. We would suggest patients receiving the COVID-19 vaccination be aware of possible ocular complications and report any symptoms, regardless of severity.

## Data availability statement

The original contributions presented in the study are included in the article/supplementary material. Further inquiries can be directed to the corresponding author.

## Ethics statement

Ethical approval was not required for the study involving humans in accordance with the local legislation and institutional requirements. Written informed consent to participate in this study was not required from the participants or the participants’ legal guardians/next of kin in accordance with the national legislation and the institutional requirements. Written informed consent was obtained from the participant/patient(s) for the publication of this case report.

## Author contributions

The authors IT and ST had contributed equally in first authorship. All authors contributed to the article and approved the submitted version.
